# MSC in Tendon and Joint Disease: The Context-Sensitive Link Between Targets and Therapeutic Mechanisms

**DOI:** 10.3389/fbioe.2022.855095

**Published:** 2022-04-04

**Authors:** Susanne Pauline Roth, Janina Burk, Walter Brehm, Antonia Troillet

**Affiliations:** ^1^ Veterinary Teaching Hospital, Department for Horses, Veterinary Faculty, University of Leipzig, Leipzig, Germany; ^2^ Equine Clinic (Surgery, Orthopedics), Justus-Liebig-University Giessen, Giessen, Germany; ^3^ Clinic for Horses, Ludwig-Maximilians-University of Munich, Munich, Germany

**Keywords:** mesenchymal stromal cells (MSC), tendon, joint, osteoarthritis, context sensitivity, target, immunoregulation

## Abstract

Mesenchymal stromal cells (MSC) represent a promising treatment option for tendon disorders and joint diseases, primarily osteoarthritis. Since MSC are highly context-sensitive to their microenvironment, their therapeutic efficacy is influenced by their tissue-specific pathologically altered targets. These include not only cellular components, such as resident cells and invading immunocompetent cells, but also components of the tissue-characteristic extracellular matrix. Although numerous *in vitro* models have already shown potential MSC-related mechanisms of action in tendon and joint diseases, only a limited number reflect the disease-specific microenvironment and allow conclusions about well-directed MSC-based therapies for injured tendon and joint-associated tissues. In both injured tissue types, inflammatory processes play a pivotal pathophysiological role. In this context, MSC-mediated macrophage modulation seems to be an important mode of action across these tissues. Additional target cells of MSC applied in tendon and joint disorders include tenocytes, synoviocytes as well as other invading and resident immune cells. It remains of critical importance whether the context-sensitive interplay between MSC and tissue- and disease-specific targets results in an overall promotion or inhibition of the desired therapeutic effects. This review presents the authors’ viewpoint on disease-related targets of MSC therapeutically applied in tendon and joint diseases, focusing on the equine patient as valid animal model.

## Introduction

The former idea behind the application of mesenchymal stromal cells (MSC) in injured tissue was to transplant a source of undifferentiated progenitor cells and thereby achieve local effects, which were deemed to be a direct integration of MSC, further leading to restoration and regeneration at the site of injury. In musculoskeletal conditions, this is supported by studies investigating the retention and fate of locally applied MSC and by the repeated proof of MSC differentiation potential into osteogenic, chondrogenic, adipogenic and myogenic cell lines (Forest et al., 2010; Sole et al., 2013; Vieira et al., 2014). However, this assumption is more and more supplemented by findings about the ability of MSC to communicate transcellularly by direct cell-to-cell contact and release of soluble factors and extracellular vesicles (Islam et al., 2012; Liu et al., 2018; Witwer et al., 2019). With the knowledge of these transcellular communication mechanisms, the idea of a locally acting MSC requires a broader explanation than the plausible cell replacement theory alone. Moreover, locally applied MSC seem to be adaptive and promote context-sensitive cell communication. To predict MSC-target interaction, the cell and matrix composition and immunological state of the target tissue are pivotal. However, due to the complex pathophysiological mechanisms in the course of inflammation, cell and matrix destruction as characteristics at the site of injury are inconsistent, depending on the type of tissue, the grade of cell destruction and the grade of local inflammatory reaction (Ackerman et al., 2021; Scanzello and Goldring, 2021). Furthermore, though not being addressed within the present work, tissue specific resident MSC have already been shown to react context-sensitive in the respective pathophysiological microenvironment and may also be relevant for target interaction of applied MSC (Costa-Almeida et al., 2019).

Animal models play a vital role in providing information about tissue-context-sensitivity of MSC. Particularly the clarification of the MSC mode of action in injured tendons and joints forced scientists to study artificially induced lesions in various species (Delling et al., 2015; Ahrberg et al., 2018; Kwon et al., 2018; Kim et al., 2019; Khan et al., 2020). Nevertheless, all artificial disease models remain an approximation and do not allow to investigate the naturally occurring pathophysiological circumstances. Therefore, we consider it crucial to include animal models based on naturally occurring tendon and joint diseases in basic research strategies, for which the horse is most suitable and repeatedly used (Becerra et al., 2013; Smith et al., 2013; Berner et al., 2016; Broeckx et al., 2019). However, the so far limited number of existing pre-clinical and clinical studies focus on clinically detectable effects and safety, thereby only indirect conclusions about target specific cellular mechanisms of action can be drawn.

Understanding the context-sensitive mode of action of MSC specifically in tendons and joints will form the basis for their future successful application. Here, we discuss selected therapeutic targets and mechanisms of MSC applications in tendon and joint diseases and illustrate their mutual interplay. Due to its high relevance with respect to investigating naturally occurring diseases, we focus on research in the equine model.

## Targets and Mechanisms of MSC in Tendon and Joint Disease

The desired targets of MSC in therapeutic applications can be deviated from the pathophysiological mechanisms driving the respective disease. However, it is important to note that the desired targets are not necessarily the targets that are actually addressed, and even if so, the effect on the target might not be as initially expected. In this line, it must be considered that MSC are highly sensitive to their environment, which will be further addressed below. Nevertheless, aiming at a well-directed cell therapy, the first step must be to identify pathophysiological key players and to observe the effects of MSC after transplantation.

### Possible Targets of MSC in Acute and Chronic Tendon Disease

Possible targets of MSC in tendon disease include cellular components on the one hand, such as the resident tenocytes, invading immune cells and endothelial cells, and the extracellular matrix (ECM) on the other hand. Regeneration of the highly specialized matrix must always be the central goal, as it is responsible for tendon function. However, a functional replacement of cells and ECM by the MSC alone is unlikely to be achieved. Rather, the MSC should support a milieu which allows the tendon to regenerate. In this line, the cellular targets could be in the foreground in phases of active inflammation and acute injury, whereas the scarred ECM may need to be focused in chronic tendon disease.

In acute tendon disease or injury, activated tenocytes and invading immune cells, namely macrophages, foster a milieu of inflammation and further matrix degradation. Indeed, in a canine model of acute tendon injury, macrophages could be identified as a target of MSC treatment, which promoted their M1/M2 phenotype switch (Shen et al., 2016; Gelberman et al., 2017). While the identification of the underlying molecular mechanisms and targets is more complex, this effect appeared to be mediated by interleukin (IL)-4 (Shen et al., 2016; Gelberman et al., 2017). The specific interplay of tenocytes and MSC *in vivo* has not been elucidated in much detail so far, yet besides direct tenocyte targeting, a protection of tenocytes *via* macrophage modulation and overall reduction of pro-inflammatory stimuli can be assumed (Manning et al., 2015)**.** Furthermore, neovascularization plays a critical and often controversially discussed role in tendon healing, as it is crucial to successful healing but persistently increased vascularization negatively impacts long-term outcomes (Korntner et al., 2019; Liu et al., 2021). A transiently increased vascularization has repeatedly been observed as a response to MSC treatment of equine tendon lesions (Conze et al., 2014; Ahrberg et al., 2018). This suggests that vascular endothelial cells are targeted in a beneficial way, which likely is related to increased vascular endothelial growth factor (VEGF) levels (Okamoto et al., 2010; Yuksel et al., 2016).

In chronic tendon disease, failed ECM repair has led to scar tissue within the tendon. Its inferior biomechanical properties predispose to re-injury and its altered biophysical and biochemical properties fail to provide the appropriate guidance for regeneration to resident cells. Therefore, it appears advantageous to target the matrix remodeling process, with enzymatic degradation of scar matrix components and promotion of collagen I fibrillogenesis and cross-linking. So far, some insights into MSC-driven matrix remodeling *in vivo* can be deduced from studies dealing with acute equine tendon disease, but suitable models for chronic disease are lacking. Here, it could not only be shown that the tendon matrix has an improved architecture and composition after MSC injection (Schnabel et al., 2009; Crovace et al., 2010; Smith et al., 2013), but also that the treatment reduced collagenase [matrix metalloproteinase (MMP)13] activity (Smith et al., 2013) and upregulated stromelysin (MMP3) gene expression (Romero et al., 2017). This might indicate a selective and beneficial support of enzymatic tendon matrix remodeling, but data are still scarce and, importantly, these results were obtained after treatment of (sub)acute lesions but not after treatment of chronic disease.

### Possible Targets of MSC in Osteoarthritis

Osteoarthritis (OA) was originally understood as a classical “wear and tear” disease, mainly related to degenerative changes in the articular cartilage. However, nowadays OA is referred to as a complex, multisystem disease affecting the whole joint and detailed pathophysiological mechanisms are still not completely understood. Nevertheless, it is beyond doubt that inflammatory processes in the synovial membrane play a crucial role in the initiation of OA and in promoting subsequent cartilage damage and pain at least in the inflammation-driven subset of patients (de Lange-Brokaar et al., 2012). Therefore, the previously suggested differentiation potential of MSC into chondrocytes aiding cartilage regeneration (Satué et al., 2019; Song et al., 2021) was pushed into the background against the ability of the MSC to target local immunocompetent cells.

OA-related synovitis is mainly driven by the innate immune system, which should therefore be targeted by MSC therapies, again with an outstanding role of macrophages. The macrophages potentially targeted by MSC include invading pro-inflammatory monocyte-derived macrophages within the synovial fluid and adjacent joint tissue as well as resident immunomodulatory synovial macrophages consisting of several subgroups of cells (van den Bosch, 2021). Besides their pivotal role in OA-related synovitis, synovial macrophages are considered critical for tissue homeostasis, thus being reasonably involved in the re-establishment of immune homeostasis within the injured joint. Recent studies in the mouse model indicate different roles of invading monocyte-derived and tissue resident synovial macrophages during homeostasis as well as disease. It was shown that locally renewing resident macrophages within the inner synovial lining form membrane-like structures as protective physical barrier between the intraarticular space and the synovial capillary network (Culemann et al., 2019). These resident synovial macrophages maintain their immune-regulatory function even within an inflammatory environment. However, the specific pathogenetic role of these cells has not yet been addressed in OA (Haubruck et al., 2021). Tracking ferumoxytol-labeled murine MSC in a model of induced OA, MSC-treated joints were reported to show a reduced number of pro-inflammatory macrophages in favor of an increased proportion of homeostatic polarized macrophages (Hamilton et al., 2019). In co-culture models, it has been shown that MSC reduce M1-like-activating factors such as IL-1β and tumor necrosis factor (TNF)-α and induce typical M2-like macrophage markers such as IL-10, cluster of differentiation (CD)163 and CD206, partially through the prostaglandin E_2_/cyclooxygenase two pathway. Since this was shown in contact as well as in trans-well cultures, the authors suggest different MSC-mediated phases of immunomodulation including firstly an interaction *via* soluble factors and secondly a direct adhesion to the synovium (Manferdini et al., 2017). Yet so far, it remains to be understood whether applied MSC rather support tissue-resident macrophages or regulate primarily the inflammatory phenotype of the invading, mainly pro-inflammatory macrophages. *In vivo* and *ex vivo* models, ideally based on naturally occurring OA, could shed more light on these MSC-macrophage interactions.

Polymorphonuclear cells could represent a further target for MSC applied during OA-related synovitis. In a murine model of induced OA of the knee, locally applied MSC attracted colocalizing polymorphonuclear cells within the synovium. This was likely due to an IL-1β-mediated increased chemokine release of the applied MSC and led to the up-regulation of the phagocytic activity as well as the down-regulation of the pro-inflammatory cytokine release of the locally clustered polymorphonuclear cells (Van Dalen et al., 2019). This upregulation of the phagocytic activity might contribute to the removal of cartilage fragments from the synovial fluid, thus breaking the vicious inflammatory circle in OA.

## Adaption of MSC to Their Targets in Pathophysiological Contexts

MSCare highly sensitive to their environment, which entails a mutual interplay between the transplanted cells and their pathologically altered targets. The latter represent a crucial part of the disease milieu that will influence the MSC once transplanted (Table 1). It remains a central question whether their adaptation to the disease milieu leads to a promotion or inhibition of the desired effects on the respective targets.

**TABLE 1:** Overview of microenvironmental factors and their effect on MSC potential mechanisms of action in tendon and joint disease.

Microenvironmental factors influencing MSC mode of action in tendon and joint diseases			
Soluble components	Cellular components	Extracellular environment	
Cytokines, chemokines, enzymes, exosomes	Damaged resident cells, invading leukocytes	Extracellular matrix composition and architecture, oxygen tension	
**Putative effects on therapeutically applied MSC in tendon and joint diseases**			
**Differentiation potential ↓**	**Immunomodulatory function ↑**	**Angiogenic effects ↑**	**Matrix remodeling ↓**
• Reduced by inflammation	• Stimulated by inflammation	• Stimulated by hypoxia and inflammation	• Reduced by fibrotic extracellular matrix
• Possible misrouted osteogenic differentiation	• Production of anti-/pro-inflammatory cytokines• Regulatory effects on cells of the adaptive and innate immune system, including macrophages	• Growth factor release• Support of endothelial cells	• Altered matrix-degrading enzyme activity

Inflammatory conditions, which are often present in tendon as well as joint disease, are well-known to impact on MSC. MSC harvested from inflammatory environments show a decreased and variable fitness, which we have demonstrated for equine synovial fluid-derived MSC from osteoarthritic joints (Burk et al., 2017). In humans, the inflammatory state of synovial fluid in OA-affected knees modulates not only the proliferation of synovial fluid-derived MSC, but also induced a reduced differentiation potential, which is suggested to result in a lower ability to reverse OA (de Sousa et al., 2019). Additionally, MSC from discarded articular cartilage collected from OA patients during joint replacement therapy showed a rapid and strong mineralization upon chondrogenic induction, while markers of chondrocyte hypertrophy and stem cell osteogenesis were induced. These complex mechanisms demonstrate that MSC differentiation within the inflammatory environment might be coupled with undesired chondrocyte hypertrophy and osteogenesis (Hu et al., 2019).

Subjecting healthy MSC to inflammatory conditions impacts on their mode of action. This includes a decreased differentiation potential but can, up to a certain extent of inflammation, promote MSC immunomodulatory and protective mechanisms. With regard to tenogenic differentiation, we have shown that not only the presence of pro-inflammatory cytokines, namely IL-1β, but also the presence of leukocytes decreased the expression of the tendon transcription factor scleraxis in equine MSC (Brandt et al., 2018). Similarly, chondrogenic differentiation in pellet culture was decreased in the presence of the pro-inflammatory cytokines (Brandt et al., 2018), corresponding to findings in human MSC (Kondo et al., 2013; Liu et al., 2017). On the other hand, interestingly, chondrogenically differentiated equine MSC responded less to IL-1β stimulation than their naïve counterparts (Bundgaard et al., 2020). However, most likely more important than differentiation, pro-inflammatory stimulation has repeatedly been shown to increase MSC immunomodulatory potential. In a co-culture model using equine MSC and stimulated or non-stimulated leukocytes, we could show that the modulatory MSC mechanisms depended on the extent of inflammatory stimulation. Mild inflammatory conditions increased the percentage of MSC synthesizing the anti-inflammatory IL-10, while stronger inflammatory conditions promoted the regulatory effects of MSC on T cells, possibly *via* prostaglandin E2. However, not all effects observed in strong inflammatory conditions were strictly anti-inflammatory (Hillmann et al., 2019). With respect to joint disease, recent data has shown that synovial fluid collected from OA-affected joints influences the immunomodulatory properties of the MSC secretome and thereby promotes an anti-inflammatory subset of immune cells including an enhanced macrophage polarization into the M2-like phenotype (Cifù et al., 2020). Hypoxic conditions strongly influenced the migration and cytokine receptor expression of MSC cultured in synovial fluid collected from OA patients (Manferdini et al., 2020).

Extracellular matrices also have a strong impact on MSC properties and behavior (Li et al., 2021). However, the effects of pathologically altered ECM on MSC are still widely unknown, despite their relevance for treating chronic fibrotic conditions, attempting to specifically target the ECM. We observed that culturing equine MSC on decellularized tendon ECM failed to display synergistic effects with the tenogenic transforming growth factor (TGF)-β3 (Roth et al., 2018). This may be due to inhibitory effects of integrin/Rho/Rho-associated protein kinase (Rho/ROCK) axis activation by the extracellular matrix on canonical TGF-β3/smad2/3 signaling (Melzer et al., 2021), providing an example of cell-ECM interactions that could interfere with assumed mechanisms. Recently, we could also demonstrate that MSC undergo pathological adaptions upon exposure to scarred ECM. When culturing equine MSC on decellularized tendon matrices obtained from tendons with naturally occurred chronic disease, tenogenic differentiation was evident despite the ECM alterations in the tendon matrix, but the gene expression and activity of MMP was decreased (Doll et al., 2021). This effect was transient, but could hamper effective targeting of the scar tissue within the ECM.

## Discussion

Our growing understanding of pathophysiology and MSC behavior will promote the development of the next generation of MSC-based therapies. However, investigating target and therapeutic cells should go hand in hand and reflects naturally occurring disease. Deciphering the mutual interplay between MSC and their targets in relevant disease environments could provide the missing link for consistent therapeutic success. Recently, an interesting step in this direction was taken with the development of a bioassay, by which the effect of a patient’s OA joint microenvironment on the ability of MSC to support cartilage formation could be deduced. MSC-based cartilage formation was modified by the OA joint microenvironment, which could be useful to predict the therapeutic outcome (Neefjes et al., 2021). Such approaches may help to identify “non-responders” in advance and eventually lead to personalized OA treatments. Nevertheless, further strategies to deal with putative non-responders will still be required.

MSC priming, a promising strategy to enhance MSC potency and efficacy, directly results from the context-sensitive nature of the MSC, and aims to train the cells for their therapeutic task by subjecting them to pathophysiological stimuli. So far, this strategy has mainly been investigated with regard to inflammatory priming or “licensing” to enhance MSC immunomodulatory potential. For example, equine bone marrow-derived MSC primed with interferon (IFN)-γ were similarly activated as by co-culture with M1-polarized macrophages (Cassano et al., 2018). As the effects of IFN-γ were more consistent and also led to a chondroprotective secretome, the authors suggested that MSC priming before transplantation could be more successful than having the MSC activated by the pathological *in vivo* environment (Cassano et al., 2018), i.e., by their cellular targets, alone. In this line, in an equine model of chemically induced OA of the radio-carpal joint, inflammatory priming with TNF-α and IFN-γ led to an increased anti-inflammatory and regulatory effect of applied MSC. However, the repeated intraarticular application of inflammatory primed allogeneic MSC resulted in a slight transient inflammatory response, possibly indicative for an increased immunogenicity of primed MSC (Barrachina et al., 2018). This must be carefully considered, along with the risk of exacerbation upon excessive stimulation, which might affect therapeutic safety of primed MSC. So far, a therapy with non-primed MSC, which naturally adapt to the disease environment, appears to be sufficient to target the immune cells in most patients and represents the safer option until more knowledge is available. However, MSC priming may help non-responding patients and this concept in general appears highly valuable for future therapies.

Aiming to further improve MSC priming approaches, it will be interesting whether priming regimes can be tailored for specific targeting of certain cell types or ECM components. With respect to tendon and OA therapies, macrophages remain the most promising cell type to modulate or alter their functional phenotype, as key regulatory cells in tendon as well as OA-related inflammation. However, little is known about the conditions of the disease environment in which MSC target inflammation and decrease the pro-inflammatory state of macrophages at their best (van den Bosch, 2021). Therefore, pre-treatment options to improve MSC efficacy by targeted influencing of tendon and joint-associated macrophages should be set on the scientific agenda. This implies optimizing MSC and target macrophage communication applicable for different disease stages, including conditions with an unfavourable environment such as the exacerbated OA-related synovitis.

We conclude that the mode of action of locally applied MSC is influenced by the cellular and molecular microenvironment at the injured site and vice versa. In this context, MSC-mediated macrophage modulation represents a key tool to positively influence inflammation in injured tendons and joints. However, a broad range of additional target cells as well as the ECM also have to be addressed. The best possible outcome for any MSC recipient will be achieved when the target tissue is characterized and the applied MSC, including potential priming, are matched with each other as specifically as possible.

## Data Availability

The original contributions presented in the study are included in the article/Supplementary Material, further inquiries can be directed to the corresponding author.
